# gPGA: GPU Accelerated Population Genetics Analyses

**DOI:** 10.1371/journal.pone.0135028

**Published:** 2015-08-06

**Authors:** Chunbao Zhou, Xianyu Lang, Yangang Wang, Chaodong Zhu

**Affiliations:** 1 Supercomputing Center, Computer Network Information Center, Chinese Academy of Sciences, Beijing, 100190, China; 2 Key Laboratory of Zoological Systematics and Evolution, Institute of Zoology, Chinese Academy of Sciences, Beijing, 100101, China; Pennsylvania State University, UNITED STATES

## Abstract

**Background:**

The isolation with migration (IM) model is important for studies in population genetics and phylogeography. IM program applies the IM model to genetic data drawn from a pair of closely related populations or species based on Markov chain Monte Carlo (MCMC) simulations of gene genealogies. But computational burden of IM program has placed limits on its application.

**Methodology:**

With strong computational power, Graphics Processing Unit (GPU) has been widely used in many fields. In this article, we present an effective implementation of IM program on one GPU based on Compute Unified Device Architecture (CUDA), which we call gPGA.

**Conclusions:**

Compared with IM program, gPGA can achieve up to 52.30X speedup on one GPU. The evaluation results demonstrate that it allows datasets to be analyzed effectively and rapidly for research on divergence population genetics. The software is freely available with source code at https://github.com/chunbaozhou/gPGA.

## Introduction

The study of speciation at the population level, or divergence population genetics, is a major focus of evolutionary research. Studies in this area typically involve sampling genes from populations and species of interest, then analyzing the patterns of genetic variation to gain insight into the processes responsible for population divergence [1]. Estimates of population parameters can be substantially improved by sampling multiple genetic markers, especially unlinked loci. Large, multilocus or genomic data sets present a rich source of information for studying population processes [2, 3].

There are many methods can be applied to population genetics analyses, such as Linkage disequilibrium, Gametic phase estimation method, Molecular diversity method and so no [4–15]. But when apply to population subdivision analyses, above methods make one of two rather extreme assumptions: (1) the diverged populations have equilibrium migration rate; (2) the diverged populations have gene flow only before they descended from a common ancestral population at some time in the past [16]. The isolation with migration (IM) model is a framework that enables the divergence time and migration rates between two populations to be estimated jointly from an alignment DNA sequences [16]. Using various parameters, IM model can capture the effects of different factors that have a role in population divergence. IM program applies the IM model to genetic data drawn from a pair of closely related populations or species based on Markov chain Monte Carlo (MCMC) simulations of gene genealogies and were originally described in reference [17]. IM program has been applied to a wide range of questions in population genetics, speciation, and hybridization [18–21]. Population parameters estimation in IM program is based on MCMC method. MCMC method is a random-walk algorithm that allows sampling from the posterior distribution [22, 23]. MCMC method is a computationally intensive method, so this places a limit on the application of IM program for population genetics analyses.

GPU is designed specifically for graphics originally. With powerful computing capacity using hundreds of processing units, General-purpose computing on graphics processing units (GPGPU) is proposed. GPGPU is the utilization of a GPU to perform computation in applications traditionally handled by the central processing unit (CPU). The dominant proprietary framework for GPGPU is CUDA which is a general purpose parallel computing platform and programming model. We can leverage the parallel compute engine in NVIDIA GPU to solve many complex computational problems in a more efficient way than on a CPU. GPU as a coprocessor of CPU is a powerful supplement for the performance of the primary processor and is popular in evolutionary biology now [24–26]. The function implementation on GPU called kernel executed *N* times in parallel using *N* different CUDA threads, as opposed to the function implementation on CPU executed only once. CUDA threads can be organized in one-dimension, two-dimension and three-dimension for different applications. CUDA threads are organized by blocks in GPU. GPU have its independent memory called global memory, and there are also shared memory, local memory, constant memory and texture memory on GPU which are similar to cache on CPU for performance promotion of different applications [27].

With strong computational power, GPU has been widely used in many fields. In this article, we present an effective implementation of IM program on one GPU based on CUDA, which we call gPGA. gPGA only implements two of the five mutation models in IM program, including Hasegawa-Kishino-Yano (HKY) model [28] and Infinite Sites (IS) model [29]. Compared with IM program, gPGA can achieve up to 52.30X speedup on one GPU. The evaluation results demonstrate that gPGA allows datasets to be analyzed effectively and rapidly for research on divergence population genetics.

## Method

### Isolation with migration model

**Fig 1:**
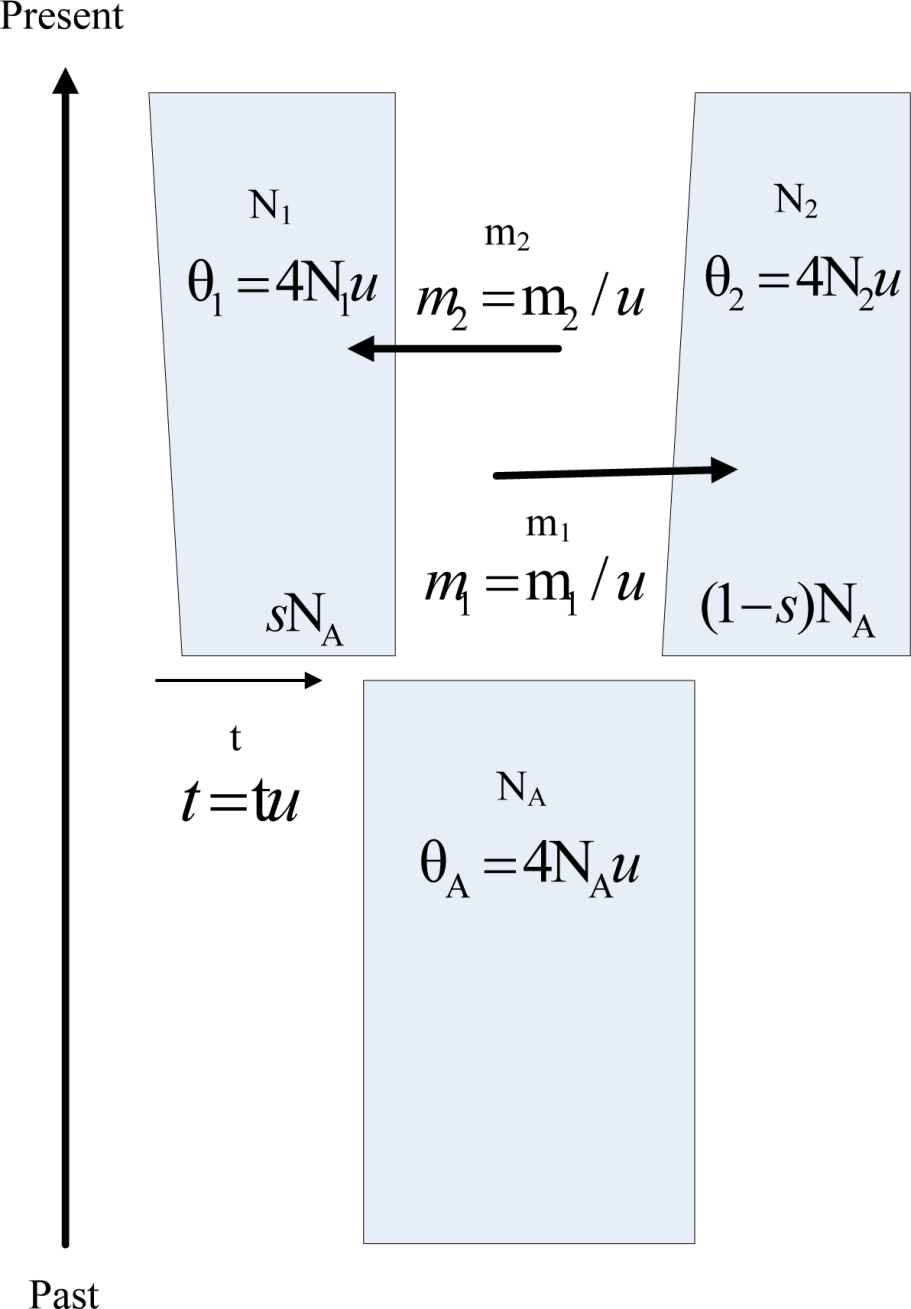
Isolation with Migration model [30]. The parameters are effective population sizes (N_1_, N_2_, and N_A_), rates of gene flow (m_1_ and m_2_), time of population divergence (t) and proportion of N_A_ that forms the founding population of N_1_ (s). So the founding sizes of the descendent populations are sN_A_ and (1-s)N_A_ respectively, where 0<s<1. Parameters are all scaled by the neutral mutation rate (u) in IM program, including θ_1_ = 4N_1_ *u*, θ_2_ = 4N_2_ *u*, θ_A_ = 4N_*A*_ *u*, *m*_1_ = m_1_ / *u*, *m*_2_ = m_2_ / *u*, *t* = *t* / *u*.

IM model includes parameters for effective population sizes (N_1_, N_2_, and N_A_), rates of gene flow (m_1_ and m_2_), time of population divergence (t) and proportion of the ancestral population that forms one of the founding populations (s) (Fig 1). In IM model, the founding sizes of the descendent populations are sN_A_ and (1-s)N_A_ respectively, where 0<s<1. Population parameters are all scaled by the neutral mutation rate (u) in IM program [30].

### MCMC method

The posterior probability of parameter is calculated through Eq 1, where D denotes sequences, T denotes phylogenetic tree, τ denotes branch lengths, and θ is the set of model parameters, where *θ* = {*θ*_1_, *θ*_2_, *θ*_*A*_, *m*_1_, *m*_2_, *t*}.

$$P(T,\tau ,\theta |D)=\frac{P(D|T,\tau ,\theta )•P(T,\tau ,\theta )}{P(D)}$$ (1)

The Metropolis-Hastings algorithm [22, 23] is used for MCMC method in IM program and works as follows: where *x* denotes the current state of the Markov chain, *x* ^’^ denotes the proposed state, *g*(*x*→*x’*) denotes the conditional probability of proposing a state *x* ^’^ given *x*.

1. randomly propose a new state *x* ^’^ according to *g*(*x*→*x’*)
2. the probability (*R*) of accepting the new state *x* ^’^ is
  $$R=min\left[1,\frac{P\left(D|x\prime \right)}{P\left(D|x\right)}\times \frac{P\left(x\prime \right)}{P\left(x\right)}\times \frac{g\left(x\prime \to x\right)}{g\left(x\to x\prime \right)}\right]$$ (2)
3. Generate a random variable *U* which is uniformly distributed on the interval (0,1). If *U*<*R*, accept the proposed state *x* ^’^. Otherwise, continue with the current state *x*.
4. Go back to step 1).

This process is repeated for a sufficiently large number of iterations until there are sufficient samples that have been drawn from Markov chain.

### Likelihood computations

**Fig 2:**
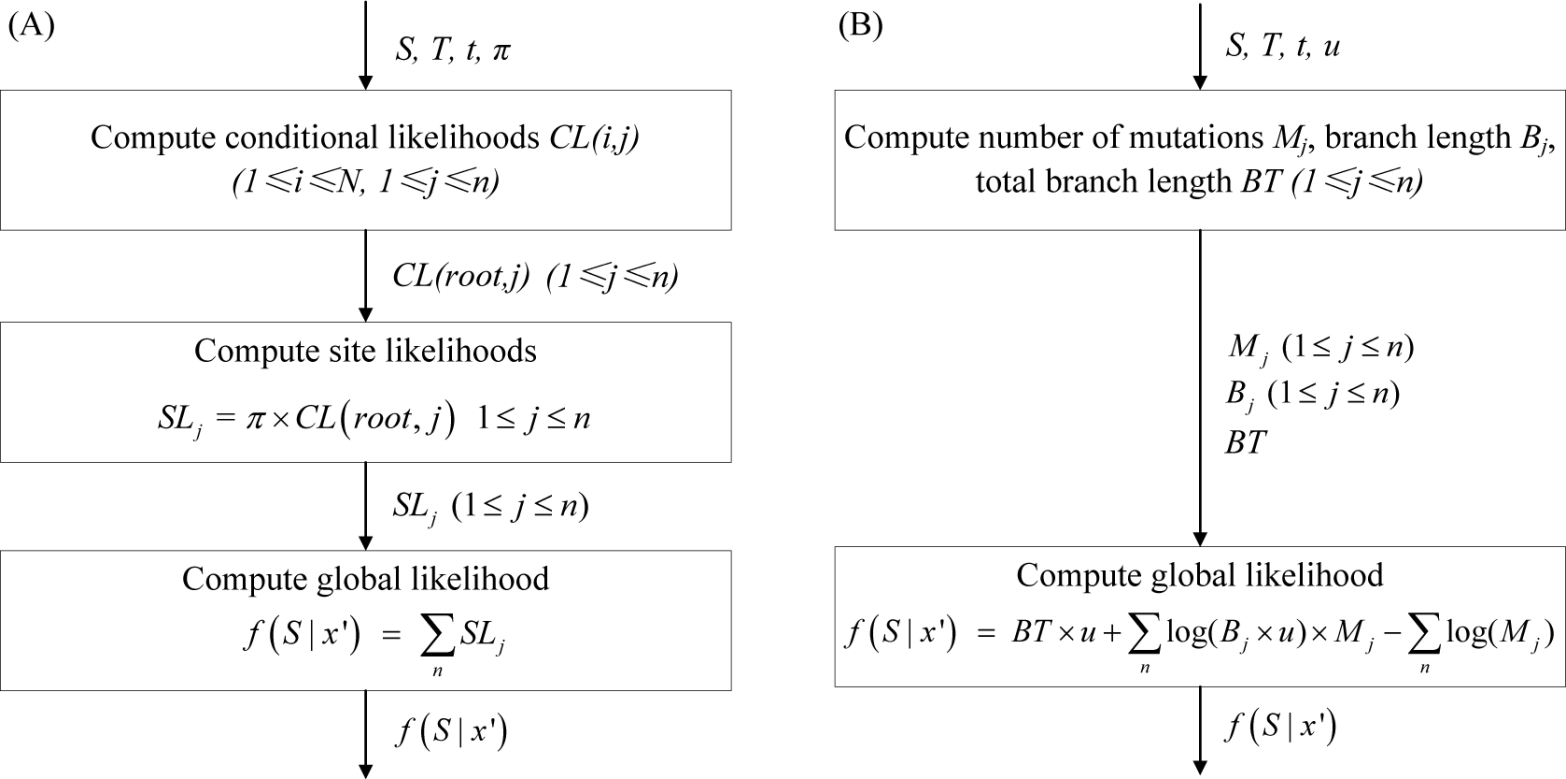
The flow chart of likelihood evaluation for HKY model and IS model. *S* denote the sequences with length *n*, *T* denote the phylogenetic tree, *t* denote the branch length, *π* denote the base frequencies of nucleotide, *u* denote neutral mutation rate, *N* denote the number of individuals for analyses. (A) *CL* denote conditional likelihood for non-leaf node in *T*, *SL* denote site likelihood for root node in *T*, *f*(*S* | *x’*) denote global likelihood. (B) *M* denote the number of mutations, *B* denote branch length, *BT* denote total branch length for *T*, *f*(*S* | *x’*) denote global likelihood.

**Fig 3:**
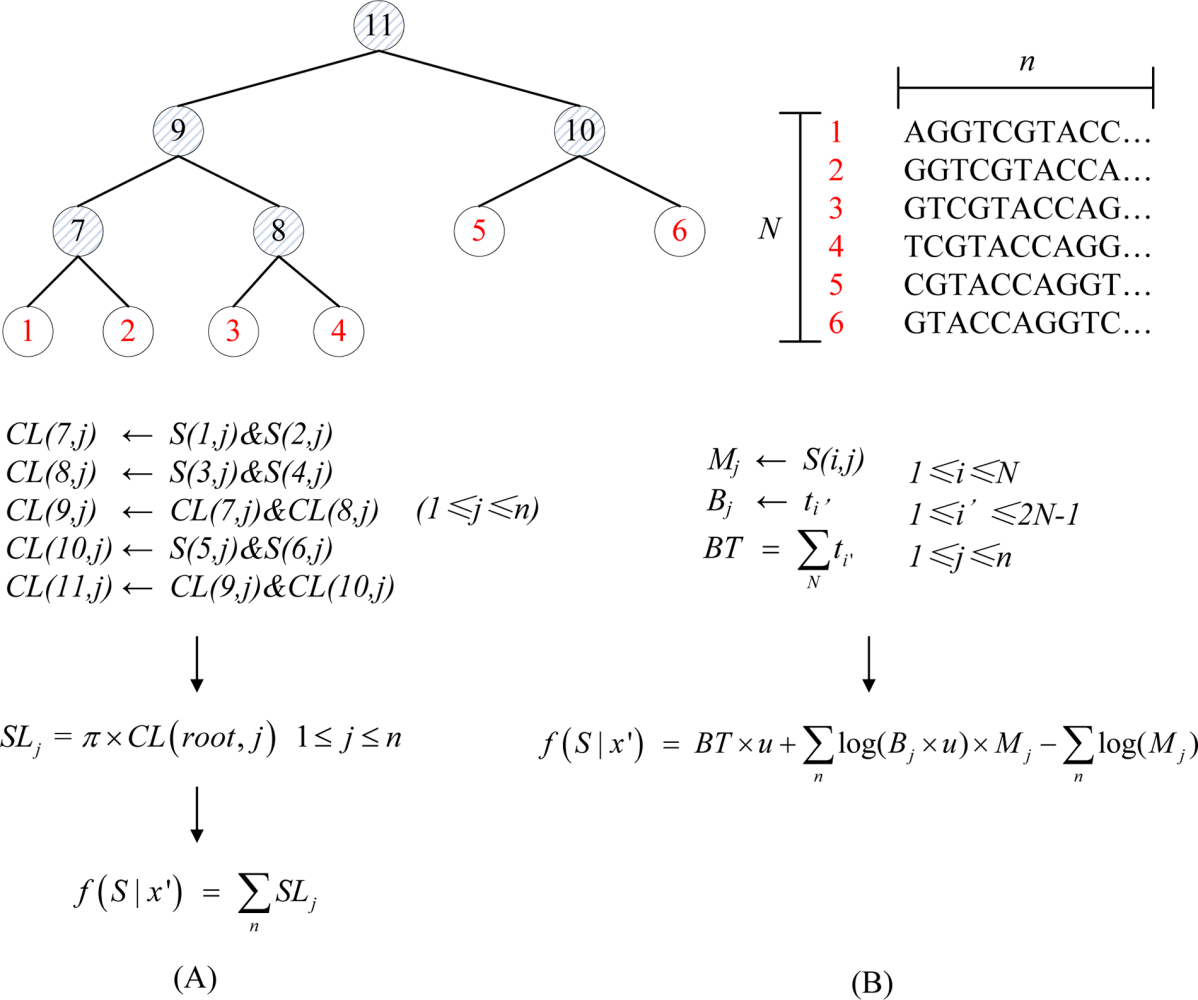
Illustration of likelihood evaluation for HKY model and IS model. *N* denote the number of individuals in population for analyses and *n* denote the length of sequences. The circles in shadow are the non-leaf nodes and the circles with red number are the leaf nodes in phylogenetic tree. (A) *S*(*i*,*j*) denote *j*th base of *i*th sequence, *CL*(*i*,*j*) denote conditional likelihood of *j*th base for *i*th node, *SL*_*j*_ denote site likelihood for *j*th base, *f*(*S* | *x’*) denote global likelihood. (B) *S*(*i*,*j*) denote the *j*th base of *i*th sequence, *M*_*j*_ denote the number of mutations of *j*th base, *B*_*j*_ denote branch length of *j*th base, *t*_*i*_ denote branch length of *i*th node, *BT* denote total branch length for phylogenetic tree, *f*(*S* | *x’*) denote global likelihood.

The likelihood evaluation is part of MCMC method and the details of likelihood computations are shown in Fig 2. When given sequences (*S*), phylogenetic tree (*T*) and branch length (*t*), the likelihood evaluation for HKY model in IM program is shown in Fig 2A. Firstly computing the conditional likelihoods (*CL*) for all the non-leaf nodes in *T*, then computing the site likelihoods (*SL*) for root node in *T*, finally computing the global likelihood (*GL*). Illustration of likelihood evaluation for HKY model is shown in Fig 3A. There are *N* = 6 individuals for analyses, and the length of *S* is *n*. The circles in shadow are the non-leaf nodes and the circles within red are the leaf nodes in *T*. Firstly for node *i* (1≤*i*≤*N*), if the descendants (*d*_*l*_,*d*_*r*_) of *i* are leaf nodes then computing *CL*(*i*,*j*) based on *S(d*_*l*_,*j)* and *S(d*_*r*_,*j)*, if the descendants (*d*_*l*_,*d*_*r*_) of *i* are non-leaf nodes then computing *CL(i*,*j)* based on *CL(d*_*l*_,*j)* and *CL(d*_*r*_,*j)* we have got already (1≤*j*≤*n*). When computing *CL*(*i*,*j*), there are 4 situations depending on its descendants (leaf nodes or not). Further, when its descendants are not leaf nodes, we can use *CL* computed last generation or *CL* computed now. So there are 9 situations for computing *CL* depending on its descendants and we define 9 GPU kernels for computing *CL* also. Then computing *SL*_*j*_ = *π* × *CL*(*root*, *j*) 1≤*j*≤*n*. Finally computing *GL* through $f\left(S|x\prime \right)\;=\;\sum_{n}SL_{j}$.

When given sequences (*S*), phylogenetic tree (*T*) and branch length (*t*), the likelihood evaluation for IS model is shown in Fig 2B. Firstly computing the number of mutations (*M*) for all sites in *S*, computing the branch length (*B*) for all sites in *S* and computing the whole branch length (*BT*). Then computing *GL*. Illustration of likelihood evaluation for IS model is shown in Fig 3B. There are *N* = 6 individuals for analyses, and the length of *S* is *n*. The circles in shadow are the non-leaf nodes and the circles within red are the leaf nodes in *T*. Firstly computing *BT* for all the nodes in *T* and for each site *j* (1≤*j*≤*n*) of *S* computing *M*_*j*_ and *B*_*j*_ through all the nodes in *T*. Only thing to note here is that the computational process breaks immediately when there are more than one mutation at a site under the topology and return 0 as *GL*. Then computing *GL* through $f\left(S|x\prime \right)\;=\;BT\times u+\sum_{n}\text{log}(B_{j}\times u)\times M_{j}-\sum_{n}\text{log}(M_{j})$.

The likelihood evaluation for HKY model and IS model are sensitive to the length of sequence through above analyses, so we focus HKY model and IS model rather than other models in IM program. From Fig 3 we also known that the computational order for node in *T* for HKY model is concerned, and this is unconcerned for IS model.

### MCMC method on GPU

Because of the different computational process for HKY and IS model in IM program, we designed different computational process for them on GPU. In order to accelerate the computation of likelihood using shared memory on GPU and reduce the communication cost between CPU and GPU, we acquire the block likelihoods (*BL*) for each block on GPU and transfer *BL* from GPU to CPU for *GL* computation. *GL* is the sum of *SL*, so we sum part of *GL* based on shared memory for each block on GPU and this is *BL*.

**Fig 4:**
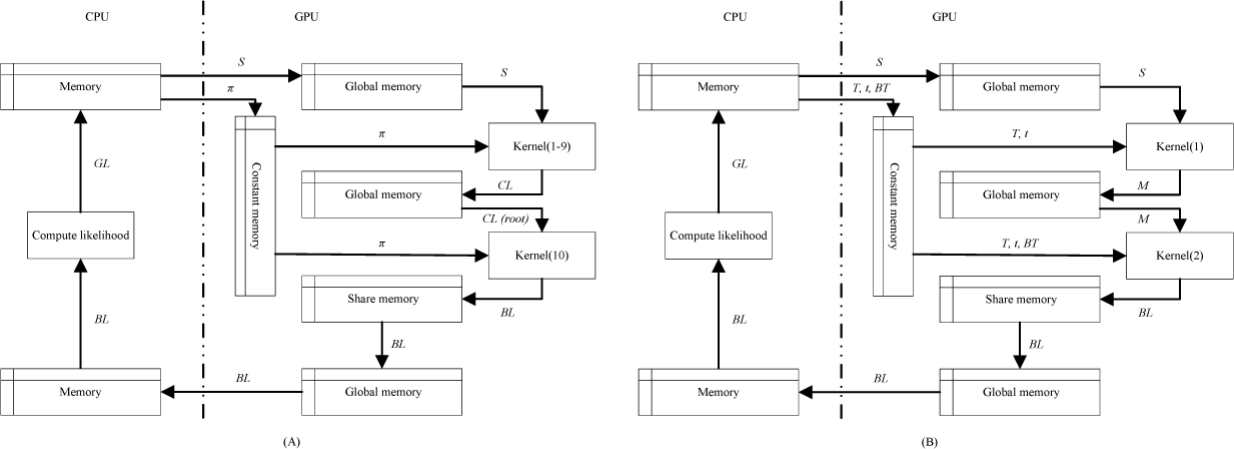
Illustration of likelihood evaluation for HKY model and IS model on GPU. CPU memory denote the RAM of computer. (A) *S* denote the sequences used for analyses, *π* denote the base frequencies of nucleotide, *CL* denote the conditional likelihood for non-leaf nodes in phylogenetic tree, *CL(root)* denote the conditional likelihood for root node in phylogenetic tree, *SL* denote the site likelihoods for root node, *BL* denote the block likelihood for root node and *GL* denote the global likelihood. (B) *S* denote the sequences used for analyses, *T* denote the phylogenetic tree, *t* denote the branch length, *M* denote the number of mutations, *BT* denote total branch length for phylogenetic tree, *SL* denote the site likelihoods for root node, *BL* denote the block likelihood for root node and *GL* denote the global likelihood.

Illustration of likelihood evaluation for HKY model on GPU is shown in Fig 4A. Considering computational order for nodes in *T*, we scheduled the order for nodes before computing likelihood on GPU. So *T* and *t* does not need to transfer to GPU. MCMC method for HKY model on GPU is as follows:

1. Initialization stage
  1.1 Allocate GPU global memory for *S*
  1.2 Transfer *S* from CPU memory to GPU global memory and transfer *π* from CPU memory to GPU constant memory
  1.3 Set *g* = 0
2. While (*g*<maximum generation) do
  2.1 Propose a new *T*
  2.2 For each non-leaf node *i* in *T* (1≤i≤*N*) do
    2.2.1 Call kernel (1–9) to compute $CL\left(i,*\right)\text{ = }\sum_{n}CL\left(i,j\right)$ on GPU and store it on GPU global memory
  2.3 Call kernel (10) to compute *SL* and *BL* using GPU shared memory and store *BL* on GPU global memory
  2.4 Transfer *BL* from GPU global memory to CPU memory
  2.5 Compute *GL* on CPU
  2.6 Accept or reject *T*

Illustration of likelihood evaluation for IS model on GPU is in Fig 4B. MCMC method for IS model on GPU is as follows:

1. Initialization stage
  1.1 Allocate GPU global memory for *S*
  1.2 Transfer *S* from CPU memory to GPU global memory
  1.3 Set *g* = 0
2. While (*g*<maximum generation) do
  2.1 Propose a new *T*
  2.2 Compute *BT* for *T*
  2.3 Transfer *T*, *t* and *BT* from CPU memory to GPU constant memory
  2.4 Call kernel (1) to compute *M*_*j*_ (1≤*j*≤*n*) using GPU shared memory and store it on GPU global memory
  2.5 Call kernel (2) to compute *BL* using GPU shared memory and store *BL* on GPU global memory
  2.6 Transfer *BL* from GPU global memory to CPU memory
  2.7 Compute *GL* on CPU
  2.8 Accept or reject *T*

When *BL* for IS model on GPU is computed completely, we continue to check the number of mutations for each site and decide to if return 0 or not.

### Communication between CPU and GPU

#### For HKY model

Sequences transfer to GPU only once. If there are *L* locus data for analyses and there are *N* individuals for each locus data with sequence length *n*, the communication cost for sequences is *L×N×n*. In each generation, MCMC method proposes a new phylogenetic tree with new branch length. However, we scheduled the nodes of phylogenetic tree before calling kernels to compute conditional likelihoods. So phylogenetic tree and branch length only exist on CPU. *π* is fixed all the time, so we transfer *π* to GPU only once. The communication cost for *π* is *L*×4. The communication cost for site likelihoods is *n*. The threads are organized by blocks on GPU. In order to reduce the communication cost, we sum the site likelihoods in each block and acquired block likelihoods. The block likelihoods are only sensitive to the number of blocks on GPU. Further the number of blocks is far less than the length of sequences.

#### For IS model

Sequences transfer to GPU only once. It is the same as HKY model, the communication cost for sequences is *L×N×n*. In each generation, MCMC method proposes a new phylogenetic tree with new branch length. So we need transfer them to GPU in each generation. The communication cost for phylogenetic tree is 3×*×*(2×*N*-1) for the node and its two descendants. The communication cost for branch length is *N*-1. It is the same as HKY model, we only transfer block likelihoods from GPU to CPU.

### Memory usage on GPU

**Fig 5:**
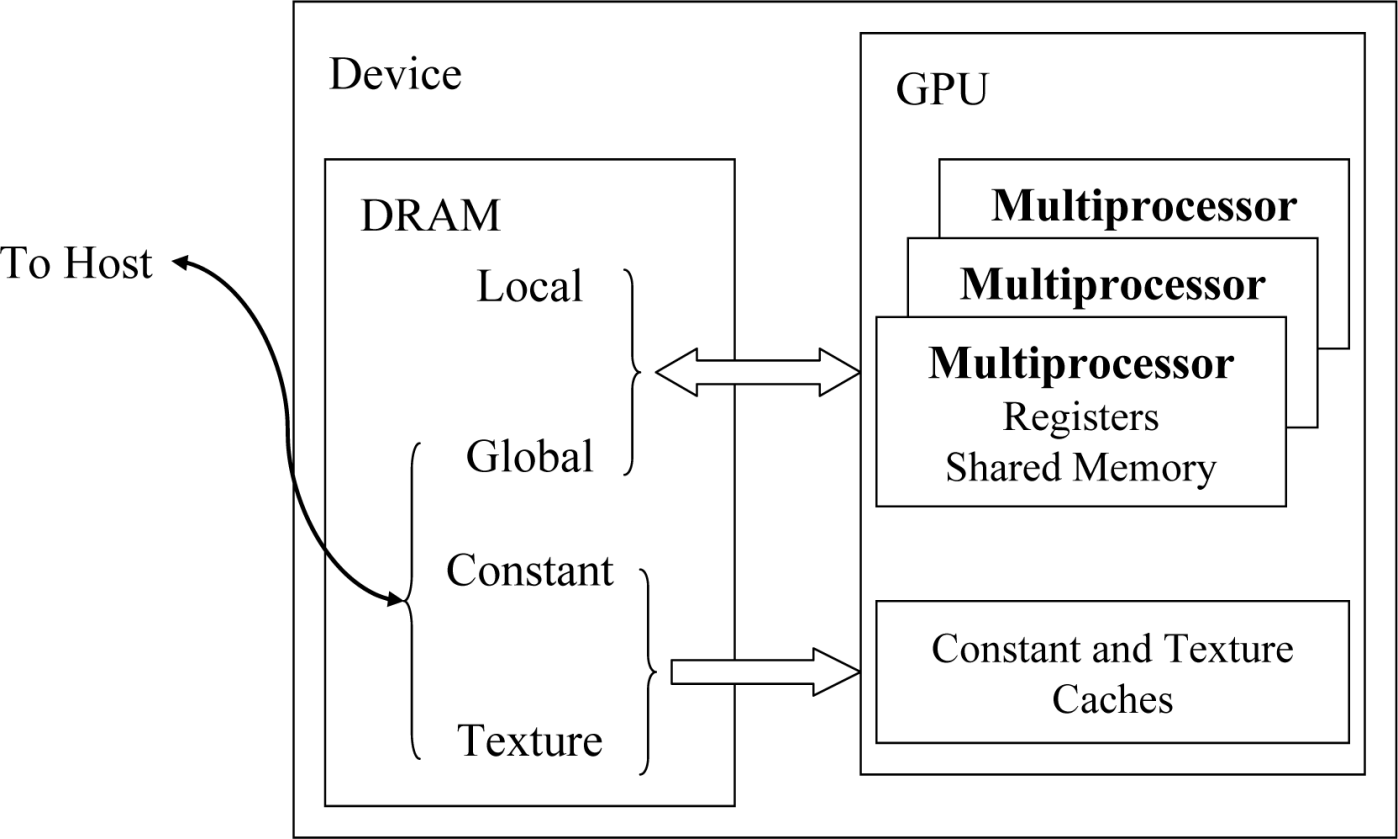
Illustration of multiple memory spaces on GPU. Global memory, local memory, constant memory, and texture memory are all no GPU card and outside GPU chip. There are caches for constant and texture memory inside GPU chip. Shared memory is shared for each multiprocessor inside GPU chip.

Illustration of multiple memory spaces on GPU is shown in Fig 5. Global memory, local memory, constant memory and texture memory are all on the GPU card. Because there are on-chip caches for constant memory and texture memory, they have much higher bandwidth and much lower latency than local memory and global memory. Shared memory is on-chip, so it has much higher bandwidth and much lower latency than local memory and global memory. For performance promotion, the constant memory and shared memory are important for gPGA. Constant memory and shared memory are all far less than global memory, so the usage of them is careful. The content in constant memory is unchanged after kernel launch, so the read-only data can store in it. The shared memory is shared for all the threads in the same block on GPU, so the block-local data can store in it.

#### For HKY model

Because memory space of sequences and conditional likelihoods are all far more than the size of shared memory and constant memory, so they are in global memory. *π* is unchanged all the time and is frequently read-only data on GPU, so *π* is in constant memory for performance promotion. Global likelihoods are the sum of site likelihoods for root node in phylogenetic tree, we sum the site likelihoods in the same block based on shared memory and acquired block likelihoods for performance promotion.

#### For IS model

Because memory space of sequences are far more than the size of shared memory and constant memory, so they are in global memory. Phylogenetic tree, branch length and total branch length are unchanged and frequently read-only data on GPU in each generation, so they are in constant memory for performance promotion. Because of the size of shared memory and constant memory is neither enough for numbers of mutations, so they are in global memory also. It is the same as HKY model, we acquired block likelihoods for performance promotion.

## Results and Discussion

We evaluated gPGA on the platform with Nvidia TESLA K20m GPU, the details are listed in Table 1.

**Table 1:** The host and device of platform.

Host		Device	
CPU	2*Intel Xeon E5-2640 (6 cores, 2.50GHz)	GPU	2*Nvidia TESLA K20m GPU
Memory	8*4GB DDR3 1333MHz	Memory	5G
Operating system	Red Hat Enterprise Linux Server release 6.2	Driver	NVIDIA Driver version 4.2

MCMC method is sensitive to the sequence length used. So we firstly simulate datasets with the same population size and different sequence length (1000, 6000, 11000, 16000) for one locus data. The simulation datasets for HKY model are simulated by Seq-Gen [31] based on the gene tree that is built by *ms* [32]. The parameters for *ms* are the same with reference [33] except the population size. The simulation datasets for IS model are simulated based on the datasets for HKY model by ourselves. All the simulation datasets are with the same population size 190, population 1 has 150 individuals and population 2 has 40 individuals.

**Fig 6:**
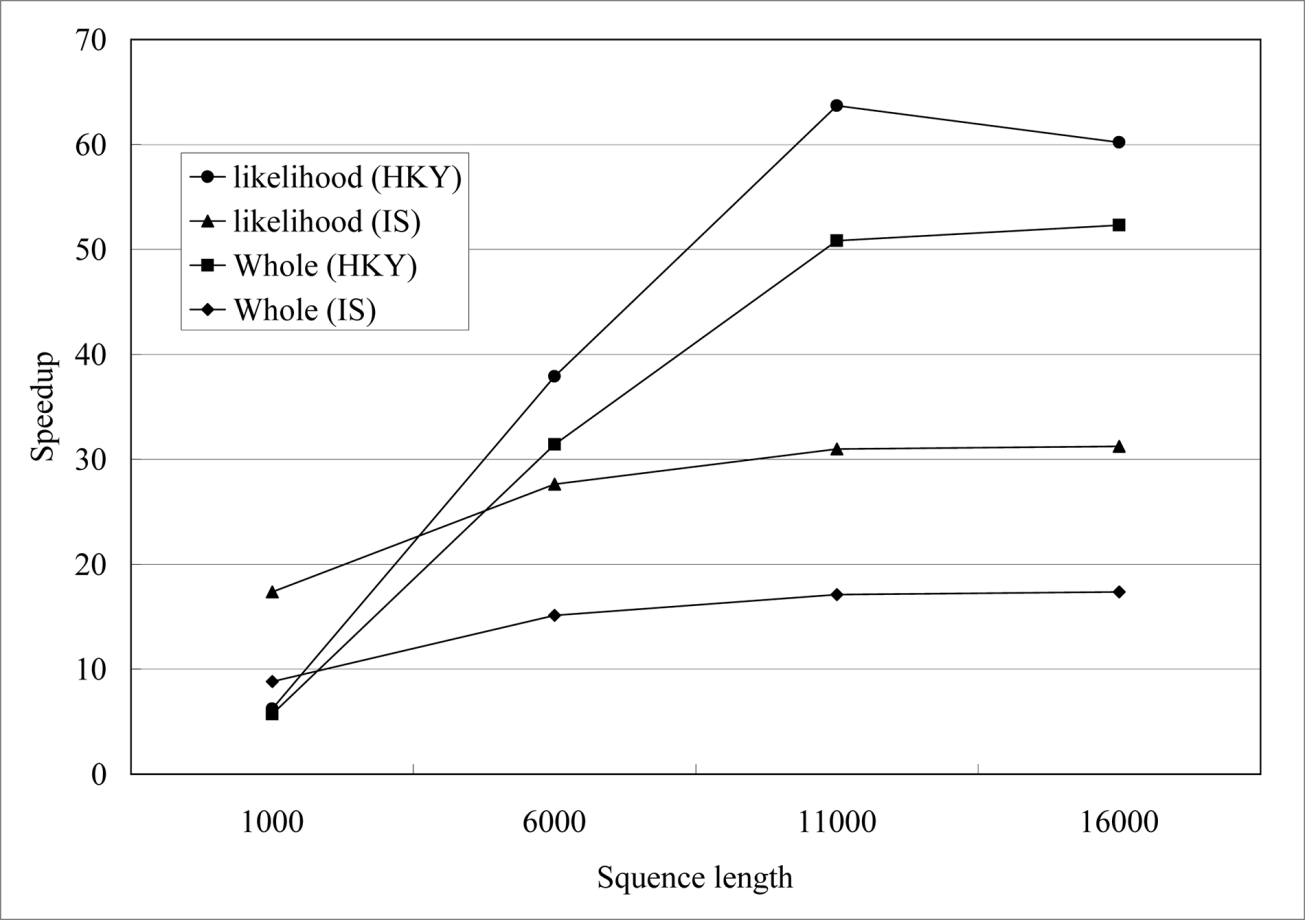
Speedups of gPGA for different sequence length *n* on GPU, *n*∊{1000, 6000, 11000, 16000}.

We performed three replicate runs of IM and gPGA for each simulation dataset and the average execution time is calculated. The burn-in generation and MCMC generation are both 10,000. The upper bounds of the population mutation parameters for population 1, population 2 and ancestor population are all 10. Maximum migration rate from population 1 to population 2 and maximum migration rate from population 2 to population 1 are all 10. Maximum time of population splitting is 10. Then we got the speedups for likelihood evaluation and whole computational process shown in Fig 6. The speedup is defined as follows:

$$S_{p}=\frac{T_{s}}{T_{p}}$$ (3)

where: *S*_*p*_ is the speedup for gPGA, *T*_*s*_ is the execution time of IM program using single CPU, and *T*_*p*_ is the execution time of gPGA using single CPU and single GPU. The implement time of different evaluation for IM program are shown in Table 2, it is the benchmark for the comparison.

**Table 2:** Implement time (Second) of different evaluation for IM program.

model	sequence length		generation		locus		Markov chain	
	likelihood	whole	likelihood	whole	likelihood	whole	likelihood	whole
HKY	409	447	409	447	409	447	808	1600
	2505	2696	2246	2450	845	922	4792	9321
	4608	4948	20763	22695	2284	2457	8882	17418
	6871	7352	204007	222711	4582	4926	13193	25782
IS	208	218	208	218	208	218	406	856
	1181	1216	1132	1166	282	298	2441	5009
	2269	2329	10759	11028	675	708	4458	9116
	3248	3333	104942	107578	1244	1309	6466	13128

Sequence length denotes implement time of different sequence length.

Generation denotes implement time of different MCMC generation.

Locus denotes implement time of different number of locus data.

Markov chain denotes implement time of different number of Markov chain.

Likelihood denotes implement time of likelihood evaluation.

Whole denotes implement time of IM program.

Fig 6 suggests that gPGA achieves increasing speedup as sequence length increases. According to Amdahl's Law, potential speedup is defined by the fraction of code that can be parallelized [34] as follows:

$$S_{p}=\frac{1}{\frac{P}{N}+S}$$ (4)

Where: *S*_*p*_ is the speedup for gPGA, *P* is fraction that can be parallelized, *S* is fraction that can not be parallelized and *N* is the number of processors used. The likelihood evaluation is the crucial computational part of IM program and it is also the fraction that can be parallelized. This is consistent with the speedups shown in Fig 6. Likelihood evaluation for HKY model is more sensitive to the sequence length than IS model. According to the description of computational process for these two model above, likelihood evaluation for IS model may break off in IM program. But likelihood evaluation for IS model in gPGA is computed completely and then we continue to check number of mutations for each site in order to break off, so gPGA slow than IM program in this situation. So in Fig 6, speedups for IS model are less than HKY model.

**Fig 7:**
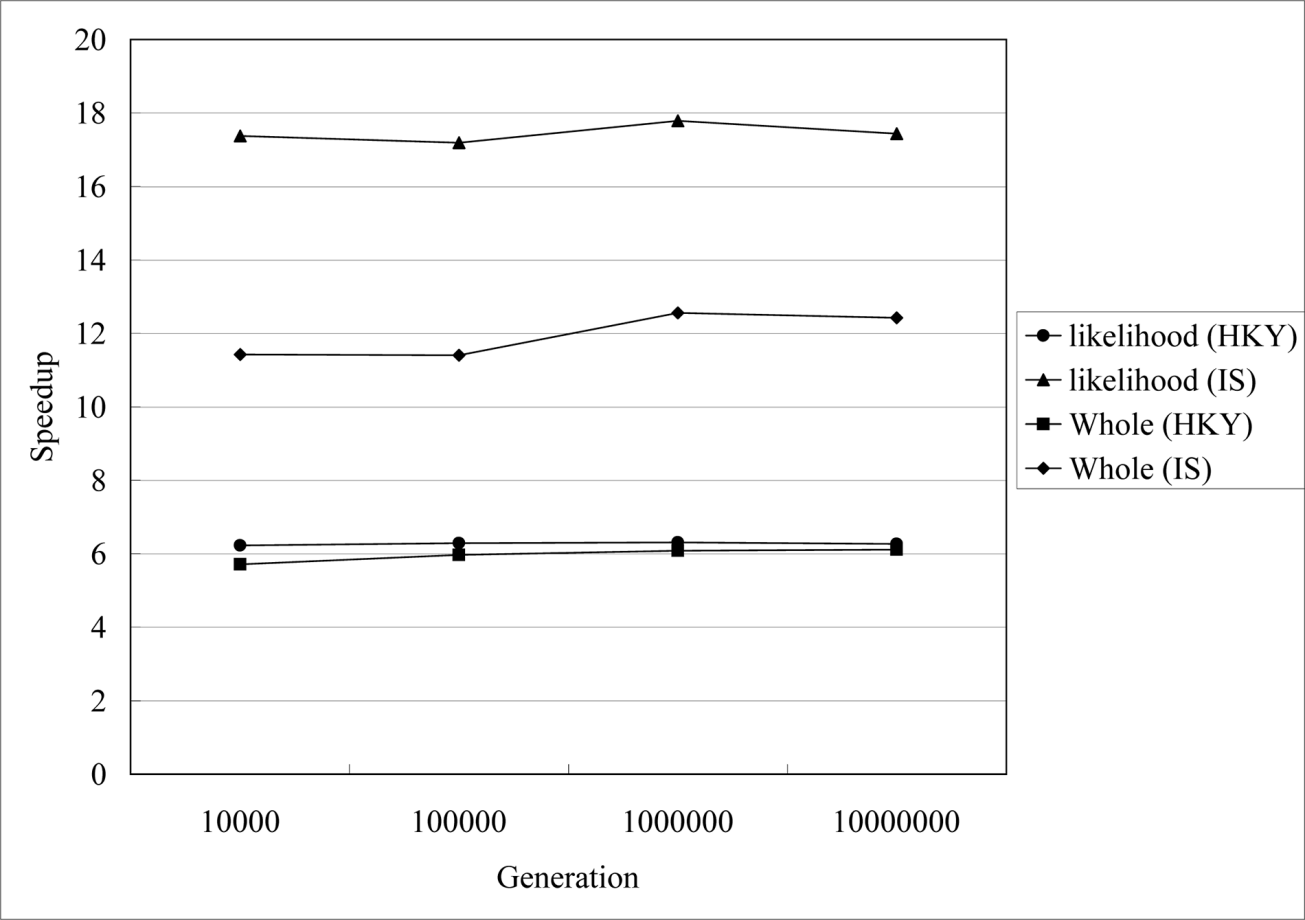
Speedups of gPGA for different MCMC generation *g* on GPU, *g*∊{10000, 100000, 1000000, 10000000}.

We secondly evaluate the influence of different MCMC generation for gPGA. The simulated datasets are that we have simulated above. They are with the same sequence length (1000) and population size (190) for one locus data. The speedups are shown in Fig 7, and the speedup is insensitive to the MCMC generation. The trends of speedups for HKY model and IS model are the same with speedups for sequence length 1000 in Fig 6. For stationary probability distribution, IM program and gPGA need sufficient MCMC generations. gPGA has stable performance when MCMC generation increasing. Performance of HKY model is more stable than IS model. This is relative to break off in likelihood evaluation for IS model we have mentioned in method section.

**Fig 8:**
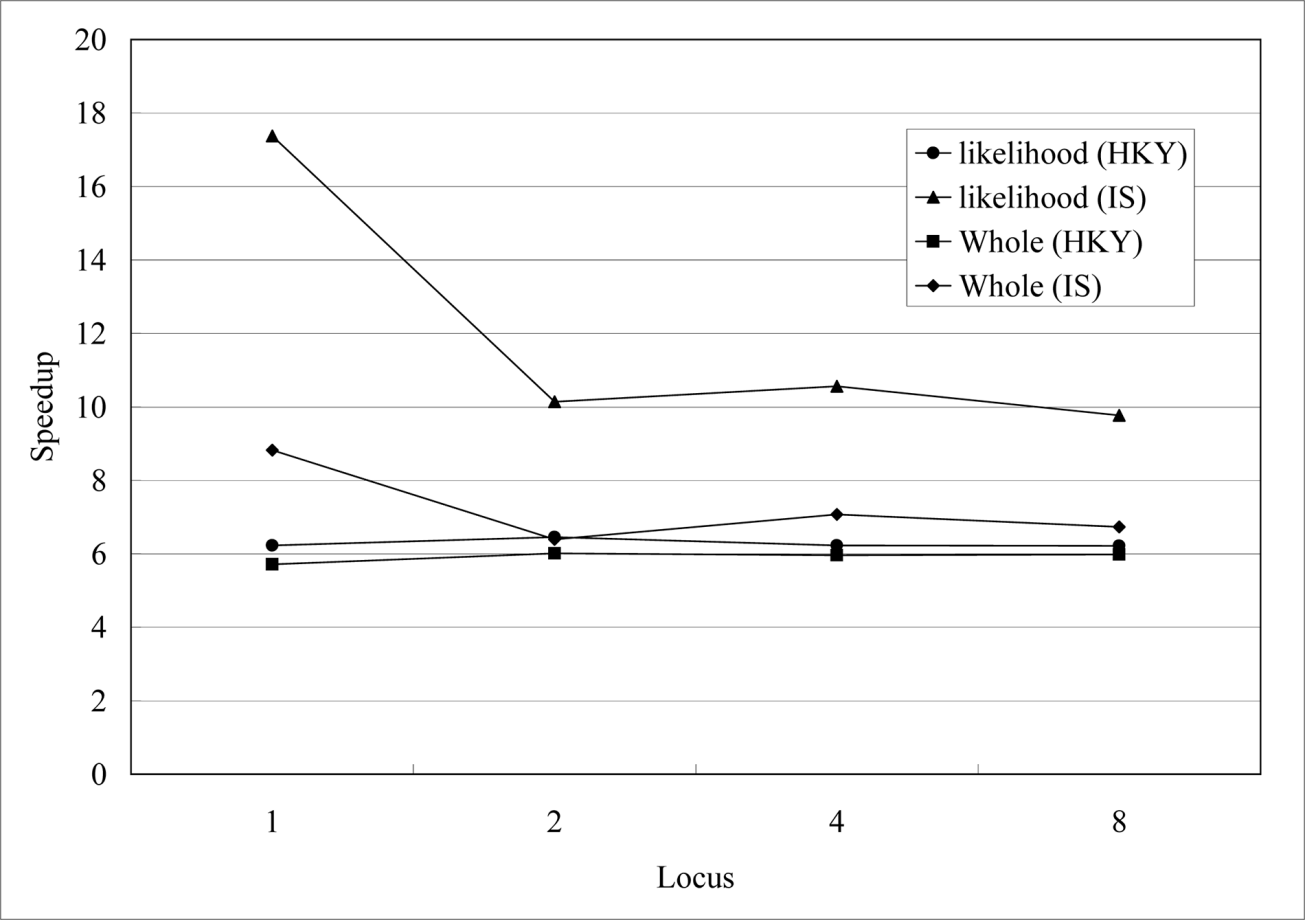
Speedups of gPGA for different number of locus data *l* on GPU, *l*∊{1, 2, 4, 8}.

We thirdly evaluate the influence of different number of locus data. The simulated datasets are combined by datasets we have simulated above. They are with the same sequence length (1000) and population size (190) for different number of locus data (1, 2, 4, 8). The speedups are shown in Fig 8, and the speedup is insensitive to the number of locus data (2, 4, 8). Speedups for IS model decline for two loci data and slightly fluctuate after that. It is the same as Fig 7, gPGA has stable performance when number of locus data increasing. Performance of HKY model is more stable than IS model. This is relative to break off in likelihood evaluation for IS model we have mentioned in method section.

**Fig 9:**
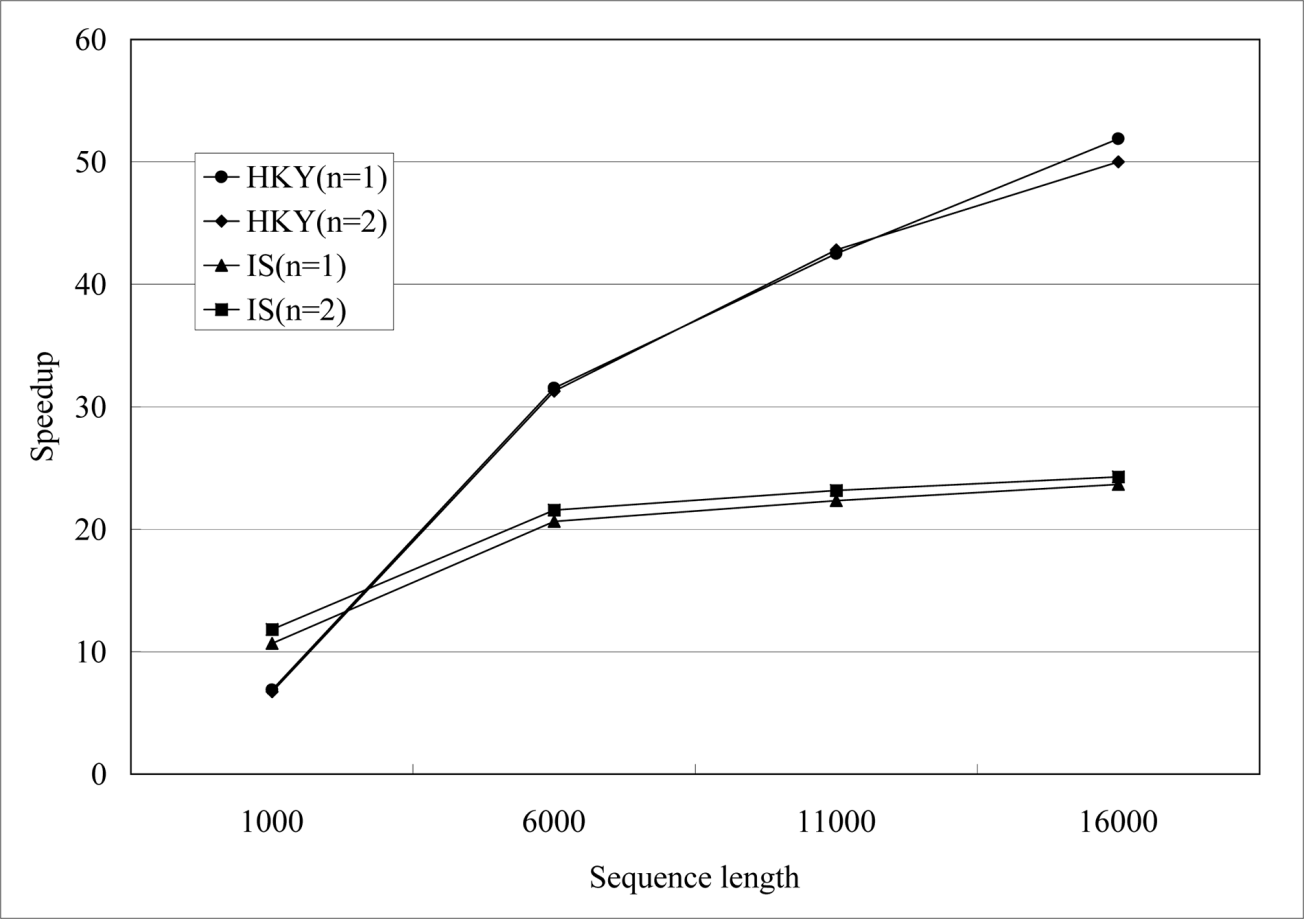
Speedups of gPGA for different number of Markov chains *m* on GPU, *m*∊{1, 2}.

To reduce the problem of sampling from local optima, the Metropolis-coupled MCMC (MC^3^) method [35] are used for IM program. The MC^3^ method implements additional Markov chains with various degrees of ‘heating’. When add one Markov chain, the computation cost for IM program will be twice. So we also evaluate the speedups for different number of Markov chains. Fig 9 suggests that gPGA achieves almost the same speedup when using two Markov chains. For good mixing and convergence, IM program and gPGA need increase number of Markov chains. In our previous work, we have applied IM program effectively on multiple CPU cores, even on cluster [36]. In future, we will apply gPGA to multiple GPUs on cluster for better speedup.

There are none optimization for IM program and the CPU code of gPGA for our evaluation. So the same amount of improvement on the realized computing time may not always be achievable with a different implementation, such as different platform used, different optimization of program and different parameters of program.

The major limitation of IM program is the restriction to samples from two populations, and it has been extended to multiple populations that have a known phylogenetic history known as IMa2 [37]. The IM-based applications are all based on bayesian inference, so the likelihood evaluation is essential. The evaluation of gPGA has shown that likelihood can be calculated efficiently on one GPU, so we have strong confidence that GPU-based implementation of IM-based applications would achieve good performance.

## Conclusions

We present an effective implementation of IM program on one GPU based on CUDA, which we call gPGA. gPGA implements two of the five mutation models in IM program, HKY model and IS model. We evaluated gPGA for different sequence length, different MCMC generation, different number of locus data and different number of Markov chains. gPGA is sensitive to sequence length, but is insensitive to MCMC generation, number of locus data and number of Markov chains. The experiments suggest that a single GPU can improve the performance of IM program by up to a factor of roughly 52.

We aim to solve the technical problems to speedup the data analyses. After examination of the additions and improvements of IMa2 program to IM program, we found the latter does not change many codes, which were found to be bottlenecks of the computation. So, we prefer to use the earlier version with simple models, parameters and distribution to focus on the efficiency. However, Dr. He, et al. [18] found results by both MPI and GPU versions fits well with what he concluded from biology. With experiences from this experiment on IM program, we will try to parallelize the IMa program or IMa2 program. Also, we will make gPGA effectively implementation on multiple GPUs including the parallelization of Metropolis-coupled chains for MC^3^ method.
